# Prevalence of diabetic kidney disease and the associated factors among patients with type 2 diabetes in a multi-ethnic Asian country

**DOI:** 10.1038/s41598-024-57723-6

**Published:** 2024-03-25

**Authors:** Kim Sui Wan, Noran Naqiah Hairi, Feisul Mustapha, Muhammad Fadhli Mohd Yusoff, Halizah Mat Rifin, Mastura Ismail, Foong Ming Moy, Noor Ani Ahmad

**Affiliations:** 1 Institute for Public Health, National Institutes of Health, Ministry of Health Malaysia, Setia Alam, 40170 Shah Alam, Selangor Malaysia; 2 Centre for Epidemiology and Evidence-Based Practice, Department of Social and Preventive Medicine, Faculty of Medicine, Universiti Malaya, 50603 Federal Territory of Kuala Lumpur, Malaysia; 3 Faculty of Public Health, Universitas Airlangga, Surabaya, 60115 East Java Indonesia; 4 Disease Control Division, Ministry of Health Malaysia, Federal Government Administration Centre, 62590 Putrajaya, Malaysia; 5 Perak State Health Department, Ministry of Health Malaysia, 30000 Ipoh, Perak Malaysia; 6 Family Health Development Division, Ministry of Health Malaysia, Federal Government Administration Centre, 62590 Putrajaya, Malaysia

**Keywords:** Chronic kidney disease, Endocrinology, Diabetes, Nephrology

## Abstract

The actual prevalence of diabetic kidney disease (DKD) in patients with type 2 diabetes (T2D) in Malaysia is unknown. We aimed to determine the prevalence of DKD and its associated risk factors among T2D patients in Malaysia. An analytical cross-sectional study was conducted using the year 2022 clinical audit dataset from the National Diabetes Registry. DKD was defined as albuminuria, a decreased glomerular filtration rate, or both. Among 80,360 patients, 62.2% were female, 68.4% were Malay, and the mean age was 61.4 years. A total of 56.7% (95% CI 56.4–57.1%) of patients were found to have DKD. Increasing age, male sex, Malay ethnicity, longer duration of diabetes, overweight, obesity, hypertension, diabetic retinopathy, diabetic foot ulcer, nontraumatic lower-extremity amputation, ischaemic heart disease, stroke, insulin, higher numbers of antihypertensive agents, antiplatelet agents, poorer HbA1c control, higher systolic blood pressure, non-achievement of triglyceride target, and non-attainment of HDL-cholesterol goal were independent risk factors associated with DKD. Clinicians, program managers, and health policymakers should target modifiable factors to manage DKD and prevent its progression to end-stage kidney disease in Malaysia.

## Introduction

Chronic kidney disease (CKD) imposes enormous disease burdens in terms of financial costs and adverse health outcomes, such as frailty, end-stage kidney disease (ESKD), decreased quality of life, and premature mortality^1^. CKD is one of the most important causes of morbidity and mortality globally^2^. This progressive condition is estimated to affect over 10% or more than 800 million of the general population worldwide^2^.

Diabetes mellitus is the main cause of CKD and ESKD in developed and developing countries^3^. CKD among patients with diabetes is known as diabetic kidney disease (DKD)^4^, and DKD is responsible for the majority of the excess cardiovascular and all-cause mortality in them^5^. Although kidney biopsy is the gold standard to diagnose DKD, it is not routinely done in real-world clinical settings^6^. DKD manifests clinically as albuminuria, decreased glomerular filtration rate (GFR), or both^4^. Estimates of DKD prevalence vary widely between nations, from 27.1% in China to 83.7% in Tanzania^5,7,8^.

Malaysia, a multi-ethnic upper-middle-income country in Southeast Asia, has the highest prevalence of diabetes in the region^9^. DKD was consistently responsible for more than 50% of ESKD among new patients requiring dialysis in Malaysia between 2011 and 2021^10^. The prevalence of DKD among type 2 diabetes (T2D) patients in the country was reported to be only 14.6% in 2019^11^. As the reported prevalence was relatively lower than that reported elsewhere across the globe^1,5^, underreporting of the condition and under-screening for DKD were postulated as potential explanations^11,12^. For perspective, the prevalence of CKD among the general adult population in Malaysia was 15.5% in 2018^13^.

Hence, a more realistic estimate of DKD prevalence in T2D patients in Malaysia is required. Moreover, in the recent World Health Organization (WHO) guidelines on the monitoring of noncommunicable diseases at health facilities, the proportion of patients with diabetes who are diagnosed with DKD is one of the recommended indicators^14^. Thus, we aimed to determine the prevalence of DKD and its associated factors among T2D patients in Malaysia.

## Methods

### Study design

An analytical cross-sectional study was conducted using the year 2022 clinical audit dataset from the National Diabetes Registry. This secondary dataset was routinely collected every year since the registry’s inception in 2009 as a surveillance platform to track the quality of diabetes care in public health clinics, aid policymaking, and used for research purposes^11^. The clinical audit, also known as the Quality Assurance Study, is under the National Quality Assurance Programme to ensure that the care given by the Ministry of Health Malaysia follows regional and international standards^11,15^.

### Study population

**Figure 1:**
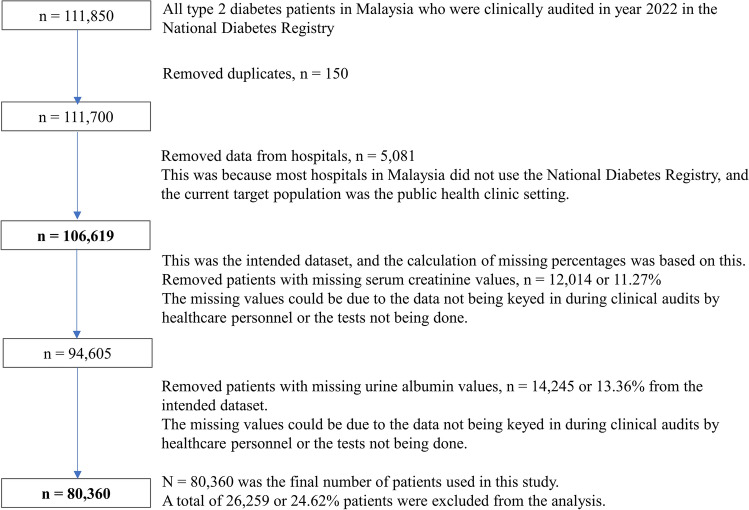
Flow diagram detailing the number of individuals at each stage of the dataset management, together with inclusion and exclusion criteria.

The target population included patients with T2D who received care from public health clinics in Malaysia. Around 70% of patients with diabetes in the country were treated in public health clinics^16^. The inclusion criteria were adult patients with T2D aged 18 years and above. Patients without documented serum creatinine or urine albumin levels were excluded. Figure 1 shows the flow diagram detailing the number of individuals at each stage of the dataset management, together with inclusion and exclusion criteria. The final number of eligible patients for analysis was 80,360. We compared the proportions of selected and non-selected patients by demographic factors in Supplementary Table S1. A higher proportion of those selected in the study were in the younger age category, females, and of Malay ethnicity.

### Dependent variable

DKD was defined as having albuminuria, a decreased GFR, or both^4^. We defined albuminuria as having macroalbuminuria and/or microalbuminuria. Macroalbuminuria and microalbuminuria were captured as categorical variables with positive or negative results, respectively, in the audit dataset. The test for microalbuminuria was routinely done in T2D patients having negative results for urine protein. Common tests used to detect albuminuria in health clinics included automated urine analysis, dipstick, 24-h urine protein, and urine albumin-to-creatinine ratio^17^.

A decreased GFR was defined as having a GFR < 60 ml/min/1.73 m^2^, according to the 2020 Kidney Disease Improving Global Outcomes (KDIGO) guidelines^18^. The GFR categories were G1, G2, G3a, G3b, G4, and G5 with GFR ≥ 90, 60–89, 45–59, 30–44, 15–29, and < 15 ml/min/1.73 m^2^, respectively^18^. Hence, GFR < 60 ml/min/1.73 m^2^ corresponded to G3a to G5. We determined the estimated GFR (eGFR) using the 2009 CKD-Epidemiology Collaboration (CKD-EPI) creatinine equation^19^, which was the preferred method for the Malaysian population^20^. A local study reported that the CKD-EPI creatinine equation was more accurate and precise than the Modification of Diet in Renal Disease (MDRD) equation^21^ The CKD-EPI equation was eGFR = 141 × min (Scr/κ, 1)^α^ × max (Scr/κ, 1)^−1.209^ × 0.993^Age^ × 1.018 (if female); Scr is serum creatinine, κ is 0.9 for males and 0.7 for females, α is − 0.411 for males and − 0.329 for females, min indicates the minimum of Scr/κ or 1, and max indicates the maximum of Scr/κ or 1^19^.

### Independent variables

The independent variables included demographic factors, comorbidities, diabetes-related complications, pharmacological treatments, and metabolic control. The demographic characteristics included age, sex, and ethnic groups. Besides the three major ethnicities (Malay, Chinese, and Indian) in Malaysia, there are indigenous people of Bumiputera Sabah and Bumiputera Sarawak who mainly reside in East Malaysia. ‘Other ethnic groups’ comprised minority ethnicities such as indigenous people of Peninsular Malaysia and those of foreign nationals. Smoking status was defined as the current smoking status.

Overweight and obesity categories were classified as having body mass index (BMI) of 23.0–27.4 and ≥ 27.5 kg/m^2^, respectively^22^. These lower cut-offs followed the World Health Organization (WHO) recommendations for Asian populations due to their greater risks of adverse cardiovascular outcomes^23^. Meanwhile, hypertension and dyslipidaemia followed clinical diagnoses or the use of corresponding pharmacological agents. Diabetes-related complications, such as diabetic retinopathy, diabetic foot ulcer, nontraumatic lower-extremity amputation, ischemic heart disease, and stroke, were based on clinical diagnoses by treating doctors.

Diabetes treatment modality was categorised as ‘lifestyle management only’, ‘oral glucose-lowering drug (OGLD) only’, ‘insulin only’, and ‘OGLD and insulin’. Hypertension treatment was categorised as zero, one, two, and ≥ three blood pressure-lowering drugs. The usage of lipid-lowering and antiplatelet agents was also described.

The metabolic control covered glycosylated haemoglobin A1c (HbA1c), blood pressure (BP), and LDL-cholesterol, the three primary treatment targets in diabetes management^20^. Most patients with T2D had a HbA1c target < 7.0%, while others, such as elderly patients and those with comorbidities, including DKD, were recommended to achieve a HbA1c goal between 7.0 and 8.0%^20^. Patients with HbA1c > 8.0% were considered to have poor glycaemic control^20^. The BP were classified according to stage I (140–159/90–99 mmHg), stage II (160–179/100–109 mmHg), and severe hypertension (≥ 180/ ≥ 110 mmHg)^24^. An additional systolic BP cut-off of 130 mmHg was used because BP < 130/80 mmHg was the individualised treatment target for patients with DKD or cardiovascular diseases^20^. Otherwise, a BP < 140/80 mmHg was aimed at those without DKD or cardiovascular diseases^20^. The LDL-cholesterol < 2.6 mmol/L was the general target, while an intensified LDL-cholesterol < 1.4 mmol/L was recommended for those with target organ damage, including DKD^20^.

The secondary treatment targets, namely triglyceride and HDL-cholesterol, were also reported^20^. The triglyceride target was < 1.7 mmol/L, and HDL-cholesterol targets were > 1.0 for males and > 1.3 mmol/L for females^20^.

### Statistical analysis

The analysis was carried out using the IBM SPSS Statistics version 23. Descriptive data were presented as frequencies with percentages for categorical variables. Whereas, mean ± standard deviation or median (interquartile range) were reported for continuous variables. The proportion of patients with DKD was presented as frequency and percentage with a 95% confidence interval. We first performed bivariate analyses. Differences between DKD status were assessed using Pearson chi-square tests for categorical variables, Student’s t-tests for means, and Mann–Whitney tests for medians. Then, multivariate binary logistic regression was carried out for independent variables with *P* values < 0.25 and clinically essential variables to determine factors associated with DKD. A forward stepwise likelihood ratio was used. The classification table, coefficient of determination, Omnibus test of model coefficients, Hosmer–Lemeshow test, and area under receiving operating characteristics (ROC) curve were reported. We also assessed multicollinearity and the interaction between variables. *P* values, adjusted odd ratios, and 95% confidence intervals were presented. The statistical significance threshold was pre-set at *P* < 0.05. All missing data were list-wise deleted in the multiple logistic regression analysis as we intend to analyse the real-world clinical data as it is. Demographic differences between patients selected in the study and those included in the multiple logistic regression analyses were compared using Chi-square tests.

### Ethical approval

This research was approved by the Medical Review and Ethics Committee of the Ministry of Health Malaysia (NMRR ID-23-01030-S6L). The Medical Research and Ethics Committee waived the requirement for informed consent because this study used secondary data without personal identifiers. All methods followed the Declaration of Helsinki and the Malaysian Good Clinical Practice Guidelines.

## Results

### Characteristics of patients

Among the 80,360 T2D patients, around 59.7% were older adults aged 60 years or older (Table 1**).** There were more females (62.2%), more Malay patients (68.4%), and more non-current smokers (94.0%). About 37.4% of the patients were diagnosed with T2D for five to ten years. The proportion of patients with overweight/obesity, hypertension, and dyslipidaemia was 84.5%, 89.4%, and 92.4%, respectively. Diabetic retinopathy (10.4%) was the most common complication, followed by ischemic heart disease (5.1%), stroke (1.8%), diabetic foot ulcers (0.9%), and nontraumatic lower-extremity amputation (0.5%). Oral glucose-lowering drugs (OGLD) were used in 91.7% of the patients, and 30.0% were on insulin therapy. About 86.7% and 87.9% of T2D patients were given antihypertensive and lipid-lowering agents, respectively. Meanwhile, 18.6% of patients were given antiplatelet agents.

**Table 1:** Characteristics of all patients, n = 80,360.

Characteristics	Total 80,360 (100.0) n (%)
Age, years	
Mean ± standard deviation	61.4 ± 11.0
18 to 59	32,420 (40.3)
60 to 69	29,234 (36.4)
70 to 79	15,639 (19.5)
≥ 80	3067 (3.8)
Sex	
Male	30,339 (37.8)
Female	50,021 (62.2)
Ethnic groups	
Malay	54,971 (68.4)
Chinese	10,423 (13.0)
Indian	5575 (6.9)
Bumiputera Sabah	5845 (7.3)
Bumiputera Sarawak	2061 (2.6)
Other ethnic groups	1485 (1.8)
Current smoking	4788 (6.0)
Duration of diabetes, years	
Median (interquartile range)	7.0 (8.0)
< 5	26,783 (33.3)
5–10	30,035 (37.4)
> 10	23,542 (29.3)
Body mass index, kg/m^2^ (n = 76,248)	
Mean ± standard deviation	28.0 ± 5.4
Underweight (< 18.5)	1224 (1.6)
Normal (18.5 to 22.9)	10,617 (13.9)
Overweight (23.0 to 27.4)	26,917 (35.3)
Obese (≥ 27.5)	37,490 (49.2)
Hypertension	71,822 (89.4)
Dyslipidaemia	74,255 (92.4)
Diabetic retinopathy	8334 (10.4)
Diabetic foot ulcer	739 (0.9)
Nontraumatic lower-extremity amputation	436 (0.5)
Ischemic heart disease	4118 (5.1)
Stroke	1414 (1.8)
Diabetes treatment modality	
Lifestyle management only	2600 (3.2)
Oral glucose-lowering drug (OGLD) only	53,692 (66.8)
Insulin only	4087 (5.1)
OGLD and insulin	19,981 (24.9)
Number of antihypertensive agents	
0	10,718 (13.3)
1	22,778 (28.3)
2	27,101 (33.7)
≥ 3	19,763 (24.6)
Lipid-lowering agents	70,640 (87.9)
Antiplatelet agents	14,976 (18.6)
HbA1c, % (n = 79,736)	
Mean ± standard deviation	7.84 ± 2.05
< 7.0%	34,836 (43.7)
7.0 to 8.0%	16,404 (20.6)
> 8.0%	28,496 (35.7)
Systolic blood pressure, mmHg (n = 79,950)	
Mean ± standard deviation	136.1 ± 15.5
< 130 mmHg	26,176 (32.7)
130 to 139 mmHg	22,362 (28.0)
140 to 159 mmHg	26,012 (32.5)
160 to 179 mmHg	4795 (6.0)
≥ 180 mmHg	605 (0.8)
Diastolic blood pressure, mmHg (n = 79,944)	
Mean ± standard deviation	77.3 ± 10.2
< 80 mmHg	45,987 (57.6)
80 to 89 mmHg	25,014 (31.3)
90 to 99 mmHg	8160 (10.2)
100 to 109 mmHg	670 (0.8)
≥ 110 mmHg	113 (0.1)
Blood pressure < 140/80 mmHg (n = 79,938)	32,612 (40.8)
Blood pressure < 130/80 mmHg (n = 79,938)	19,928 (24.9)
LDL-cholesterol, mmol/L (n = 71,037)	
Mean ± standard deviation	2.82 ± 1.11
< 2.6 mmol/L	32,550 (45.8)
< 1.4 mmol/L	3915 (5.5)
Triglyceride, mmol/L (n = 78,989)	
Mean ± standard deviation	1.66 ± 1.00
< 1.7 mmol/L	49,601 (62.8)
HDL-cholesterol, mmol/L	
Mean ± standard deviation (overall) (n = 71,422)	1.33 ± 0.40
Mean ± standard deviation (males) (n = 26,706)	1.22 ± 0.38
Mean ± standard deviation (females) (n = 44,716)	1.39 ± 0.41
> 1.0 mmol/L for males and > 1.3 mmol/L for females	41,892 (58.7)

The mean HbA1c was 7.8 ± 2.1%, and 35.7% of patients had poor glycosylated haemoglobin A1c (HbA1c) > 8.0%. The mean blood pressure (BP) was 136/77 mmHg, and 59.2% and 75.1% of the patients had BP ≥ 140/80 mmHg and ≥ 130/80 mmHg, respectively. The respective mean LDL-cholesterol, triglycerides, and HDL-cholesterol levels were 2.8, 1.7, and 1.3 mmol/L. The respective achievement of triglyceride and HDL-cholesterol targets were 62.8% and 58.7%. About 45.8% and 5.5% of patients attained LDL-cholesterol goals < 2.6 and < 1.4 mmol/L, respectively.

### Prevalence of diabetic kidney disease

**Figure 2:**
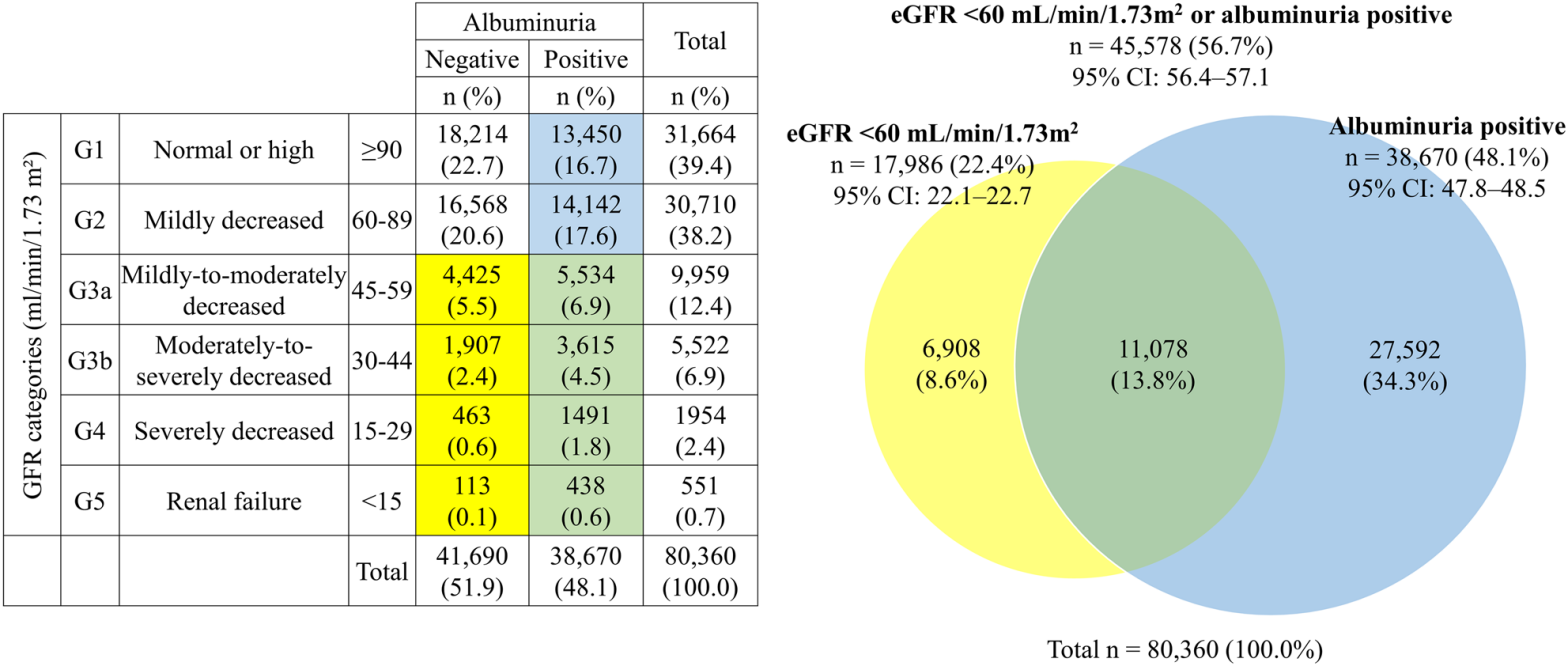
Prevalence of diabetic kidney disease based on glomerular filtration rate and albuminuria. eGFR: estimated glomerular filtration rate.

A total of 56.7% of patients were found to have DKD **(**Fig. 2**).** The Venn diagram shows that 22.4% of patients had eGFR < 60 mL/min/1.73 m^2^, while 48.1% had positive albuminuria. Around 13.8% of patients had both eGFR < 60 mL/min/1.73 m^2^ and positive albuminuria. About 8.6% of the patients had non-proteinuric kidney disease, while 34.3% had albuminuria with a normal eGFR. Regarding DKD stages, 12.4%, 6.9%, 2.4%, and 0.7% of patients were at stages G3a, G3b, G4, and G5, respectively.

### Characteristics of patients with diabetic kidney diseases

Patients with DKD were significantly older, male, of Malay ethnicity, current smokers, and had been diagnosed with diabetes for a longer duration (Supplementary Table S2). Patients with DKD generally had a higher mean body mass index (BMI), and a higher proportion of them were underweight and obese. DKD was associated with all the comorbidities and complications studied. More patients had hypertension, dyslipidaemia, diabetic retinopathy, diabetic foot ulcers, nontraumatic lower-extremity amputation, ischemic heart disease, and stroke. Correspondingly, a higher proportion of patients with DKD were treated with insulin, antihypertensive, lipid-lowering agents, and antiplatelet agents.

Patients with DKD had higher mean HbA1c levels. Despite having a less stringent HbA1c target, 40.5% had uncontrolled HbA1c levels > 8.0%. Meanwhile, a higher proportion of patients with DKD had high systolic and diastolic BP. Thus, lower proportions achieved BP < 140/80 mmHg and < 130/80 mmHg. Among those with DKD, only 10,807 (23.9%) patients achieved a more stringent treatment goal of < 130/80 mmHg.

A higher proportion of patients with DKD had LDL-cholesterol < 2.6 and < 1.4 mmol/L. Among the patients with CKD, only 6.0% achieved a more stringent LDL-cholesterol target of < 1.4 mmol/L. Patients with DKD had a higher mean triglyceride level, with a correspondingly lower proportion attaining the target triglyceride level. Meanwhile, patients with DKD had a lower mean HDL-cholesterol level with a lower proportion achieving the HDL-cholesterol goal.

### Factors associated with diabetic kidney disease

Eighteen independent factors were associated with DKD, as shown in Table 2. Increasing age, longer duration since diabetes diagnosis, overweight, obesity, hypertension, diabetic retinopathy, diabetic foot ulcer, nontraumatic lower-extremity amputation, ischaemic heart disease, stroke, insulin (alone and in combination with oral glucose-lowering drugs), higher numbers of antihypertensive agents, antiplatelet agents, poorer HbA1c control, and higher systolic BP categories were associated with higher odds ratios for DKD. In contrast, females and patients attaining triglyceride and HDL targets were less likely to have DKD. There were ethnic variations; Chinese, Indian, Bumiputera Sarawak, and other ethnicities had lower adjusted odds ratios than the Malay ethnic group.

**Table 2:** Factors associated with diabetic kidney disease.

Characteristics	Adjusted OR	95% CI for adjusted OR	*P* values
Age groups, years			
18 to 59	1.00		
60 to 69	1.20	1.16–1.25	< 0.001
70 to 79	1.91	1.82–2.01	< 0.001
≥ 80	3.15	2.84–3.50	< 0.001
Sex			
Male	1.00		
Female	0.75	0.73–0.78	< 0.001
Ethnic groups			
Malay	1.00		
Chinese	0.84	0.80–0.88	< 0.001
Indian	0.66	0.62–0.71	< 0.001
Bumiputera Sabah	1.05	0.98–1.12	0.160
Bumiputera Sarawak	0.65	0.58–0.74	< 0.001
Other ethnic groups	0.80	0.71–0.90	< 0.001
Duration of diabetes, years			
< 5	1.00		
5–10	1.04	1.00–1.08	0.041
> 10	1.27	1.21–1.33	< 0.001
Body mass index categories, kg/m^2^			
Underweight (< 18.5)	1.30	1.13–1.48	0.001
Normal (18.5 to 22.9)	1.00		
Overweight (23.0 to 27.4)	1.06	1.01–1.12	0.018
Obese (≥ 27.5)	1.21	1.15–1.27	< 0.001
Hypertension	1.28	1.14–1.43	< 0.001
Diabetic retinopathy	1.19	1.13–1.26	< 0.001
Diabetic foot ulcer	1.80	1.45–2.24	< 0.001
Nontraumatic lower-extremity amputation	1.57	1.14–2.15	0.005
Ischemic heart disease	1.10	1.02–1.20	0.014
Stroke	1.15	1.01–1.31	0.041
Diabetes treatment modality			
Lifestyle management only	1.00		
Oral glucose-lowering drug (OGLD) only	1.09	0.99–1.20	0.089
Insulin only	2.78	2.44–3.17	< 0.001
OGLD and insulin	1.46	1.31–1.62	< 0.001
Number of antihypertensive agents			
0	1.00		
1	1.22	1.09–1.35	< 0.001
2	1.56	1.41–1.73	< 0.001
≥ 3	2.03	1.82–2.25	< 0.001
Antiplatelet agents	1.14	1.09–1.19	< 0.001
HbA1c, %			
< 7	1.00		
7 to 8	1.10	1.05–1.15	< 0.001
> 8	1.29	1.24–1.34	< 0.001
Systolic blood pressure, mmHg			
< 130	1.00		
130 to 139	1.00	0.96–1.04	0.978
140 to 159	1.10	1.06–1.15	< 0.001
160 to 179	1.40	1.29–1.51	< 0.001
≥ 180	1.93	1.56–2.40	< 0.001
Triglycerides, < 1.7 mmol/L	0.73	0.71–0.76	< 0.001
HDL-C, > 1.0 for males and > 1.3 for females	0.84	0.81–0.87	< 0.001

The number of patients included in this analysis was 67,368, with 12,992 (16.2%) not included. The model was valid because the Hosmer and Lemeshow test was insignificant, *P* = 0.08. The model improved over the baseline model, as the Omnibus test was significant, *P* < 0.001. The coefficient of determination (R^2^) was 0.122, and the overall correct percentage was 63.0% in the classification table. The area under the receiving operating characteristics (ROC) curve was 0.673 (95% CI 0.669–0.677), *P* < 0.001.

HbA1c: glycosylated haemoglobin A1c; HDL-C: HDL-cholesterol; OGLD: oral glucose-lowering drug.

Supplementary Table S3 compares the demographic characteristics of included and non-included patients in the multiple logistic regression analysis. A higher proportion of those included were in the younger age category, females, and of Malay ethnicity.

## Discussion

More than half of our patients with T2D had DKD, and this prevalence falls between the 27.1% to 83.7% range reported worldwide^5^. This wide range of prevalence could be due to differences in DKD definition, patient profiles, healthcare settings, and health systems^5,25^. Our result closely approximates the global prevalence (56%) reported in the DEMAND (Developing Education on Microalbuminuria for Awareness of renal and cardiovascular risk in Diabetes) study^1,26^. Moreover, the composition of DKD by reduced eGFR and positive albuminuria among our patients is also similar to the global average; most DKD diagnoses are due to albuminuria with normal eGFR, followed by reduced eGFR and positive albuminuria, and finally, non-proteinuric kidney disease^1,26^. Indeed, a review article reported that non-proteinuric rather than proteinuric kidney diseases are the leading cause of ESKD, and non-proteinuric DKD prevails over the proteinuric form among T2D diabetes^27^.

Our prevalence of DKD is also comparable with 53% reported in a Singaporean study among a multi-ethnic group of primary care patients with T2D^25^. Again, the composition of DKD is similar to ours: 21% of their patients had reduced eGFR, while 48% had albuminuria^25^. The breakdown by GFR categories from stage G3a to G5 was also comparable with our study^25^. Besides that, a similar prevalence of reduced GFR and prevalence by GFR stages were reported in northern Thailand among T2D patients in the primary care setting^28^.

Overall, DKD is a common complication in patients with T2D in Malaysia. These results have important clinical and public health implications. Clinically, it implies the need to manage patients more aggressively to prevent the progression to ESKD, especially since diabetes was already the main contributor to dialysis in Malaysia^10,20^. DKD screening activities must also be intensified urgently, particularly because many patients in early DKD are asymptomatic and called the ‘silent majority’^6^. The clinical practice guidelines recommend screening for DKD during the initial visit and annually thereafter^20^. The lack of adherence to these recommendations constitutes clinical inertia that must be managed appropriately^29^. The WHO has recently recommended monitoring the proportion of diabetes patients with DKD in healthcare facilities, which should be considered in our health clinic settings to ensure optimal patient and programme monitoring^14^.

From a public health standpoint, the high DKD burden should alert health policymakers and programme managers about the substantial financial costs and adverse health outcomes associated with the disease, such as frailty, ESKD, reduced quality of life, and premature deaths^1^. The problem will only be magnified if more Malaysians develop diabetes, and if diabetes control remains unattainable among existing patients, more of them will end up having ESKD^20^. Around 106,000 dialysis patients are projected in Malaysia by the year 2040^30^, and the relative high share of ESKD health expenditure in the public sector will stress the financing mechanism of the disease^31^. Therefore, primary, secondary, and tertiary prevention of diabetes must be optimised to tackle the diabetes epidemic in Malaysia. This effort is consistent with the National Action Plan for Healthy Kidneys 2018–2025, a strategic plan to decrease CKD burdens in the country^32^.

Established risk factors for DKD can be divided into non-modifiable and modifiable risk factors^1,5^. Our study findings are consistent with increasing age, male sex, ethnicity, and long duration of diabetes as non-modifiable factors associated with DKD^1,5^. Previous studies reported ethnic differences with Asian, Hispanic, and indigenous Australians tend to have a higher prevalence of DKD than Caucasians^1^. Further, Asian participants were found to have the highest proteinuria compared to Hispanic, African, Caucasian, and other ethnic groups^26^. The reasons for ethnic variations in DKD are complex and multifactorial^1^. The factors include genetic factors and developmental programming, age of T2D onset, lifestyle factors, socioeconomic disadvantages, access to and uptake of care, inadequate screening rates, and poorer attainment of treatment targets^1^.

Our multi-ethnic populations in Malaysia confer an advantage in observing ethnic variations in the prevalence of DKD. However, it is unclear why the Malay ethnicity is more likely to be associated with DKD. Further research is recommended to investigate the underlying reasons for the observed ethnic differences in Malaysia.

Obesity, hypertension, poor glycaemic control, poor blood pressure control, and lipid abnormalities are known modifiable risk factors for DKD, and our study again showed consistent findings^1,5^. These results further emphasise the crucial need to optimise body weight and control metabolic targets among T2D patients. However, most of our patients with DKD did not achieve HbA1c, intensified BP, or intensified LDL-cholesterol goals. This is alarming because inadequate metabolic control can lead to the progression of DKD to ESKD^1^. Moreover, poor control of these risk factors will increase the competing risk of premature mortality, mainly due to cardiovascular diseases^1^.

We also found that diabetic retinopathy, diabetic foot ulcer, nontraumatic lower-extremity amputation, ischaemic heart disease, stroke, insulin, antiplatelet agents, and higher numbers of antihypertensive agents were associated with DKD. All these factors are proxies for more severe diabetes conditions and are clinically logical to be related to DKD. Similar associations have been reported in other epidemiological studies among patients with T2D in Singapore, Thailand, Hong Kong, and Italy^25,28,33,34^. Interestingly, we found that underweight patients were independently associated with DKD; the adjusted odds ratio was even higher than that for obesity. A Korean nationwide cohort study reported that the underweight BMI category was an independent risk factor for ESKD among patients with diabetes^35^. Moreover, those with weight loss > 10% had the fastest decline in kidney function^35^. Some plausible mechanisms include sarcopenia and oxidative DNA damage associated with weight loss^35^.

We acknowledge missing data as a limitation in this registry-based study. We noted that a higher proportion of patients selected for this study and those included in the regression analysis were females, in the younger age category, and of Malay ethnicity, and this could cause selection bias and distort the actual prevalence of DKD in this study. As increasing age and male sex are established non-modifiable risk factors for DKD^1,5^, our study sample could have pulled the DKD prevalence downwards. In other words, the actual prevalence of DKD among T2D patients in Malaysia could be higher if more males and older patients were included. In contrast, since we found that the Malay ethnicity was more likely to have DKD, a higher proportion of Malay patients could have pushed the prevalence upwards. Nevertheless, our current prevalence estimate is still valid based on available real-world clinical data, with the prevalence falling between the range reported worldwide and similar to our neighbouring countries in Singapore and Thailand^5,25,28^.

This study has other limitations. The cross-sectional design disallows any causal inference between DKD and the associated factors. Measurement errors can occur because of the lack of standardisation mechanisms between health clinics across Malaysia. Nevertheless, the data reflect real-world clinical scenarios. Besides that, the analysis was limited to the data available in the National Diabetes Registry. Uncaptured information, such as socioeconomic disadvantages, family history of DKD, previous episodes of acute kidney injury, and inflammatory markers, all associated with DKD, could not be investigated in this study^5^. We were also unable to quantify the level of albuminuria as the details were not captured in the database. Our study definition of DKD was based on a single positive reading of albuminuria, a decreased GFR, or both with no repeated measurement typically taken 3 months apart to confirm the chronic nature of CKD^18^. Nevertheless, our results are still valid with similar definitions employed in other cross-sectional studies with no repeated measures to determine CKD or DKD prevalence^25,28,36,37^. Finally, the study population was confined to T2D patients in public health clinics; hence, the results cannot be generalised to patients treated in hospitals and those with type 1 diabetes.

To our best knowledge, this nationwide study is among the first to report the prevalence of DKD and its associated factors among patients with T2D in Malaysia. Real-world clinical information offers the added advantage of depicting the actual situations in the field. Our results have established a baseline prevalence of DKD among T2D patients in Malaysia, which may aid in monitoring the WHO indicator for DKD^14^. This study uncovers a high prevalence of DKD with important clinical and public health implications, as discussed above. We hope the results will help inform policymaking and the development of clinical practice guidelines in the country. This study also benefitted from the multi-ethnic population in Malaysia. The observed ethnic differences in the prevalence of DKD may provide an impetus for new studies on kidney complications among multi-ethnic diabetes patients to improve clinical outcomes.

In summary, DKD is highly prevalent among T2D patients in Malaysia. Increasing age, male sex, Malay ethnic group, longer duration of diabetes, overweight, obesity, hypertension, diabetic retinopathy, diabetic foot ulcer, nontraumatic lower-extremity amputation, ischaemic heart disease, stroke, insulin, higher numbers of antihypertensive agents, antiplatelet agents, poorer HbA1c control, higher systolic BP, non-achievement of triglyceride target, and non-attainment of HDL-cholesterol goal are independent factors associated with DKD. Clinicians, program managers, and health policymakers should target these modifiable factors to manage DKD and prevent its progression to ESKD.

### Supplementary Information

Supplementary Table S1.

Supplementary Table S2.

Supplementary Table S3.

## Data Availability

The National Diabetes Registry dataset retrieved and analysed in this study is not available publicly due to local ethics regulation and could be obtained via written permissions to the Director General of Health, Malaysia.
