# Supplementary material for: Prevalence of diabetic kidney disease and the associated factors among patients with type 2 diabetes in a multi-ethnic Asian country
Source: Sci Rep. 2024 Mar 25;14:7074. doi: 10.1038/s41598-024-57723-6 (PMC10963363; doi:10.1038/s41598-024-57723-6)
Supplement: Supplementary file 1 — Supplementary Table S1. [file 41598_2024_57723_MOESM1_ESM.docx]

**Supplementary Table S1: Demographic comparisons between selected and non-selected patients in this study**

| **Characteristics** | **Total**  **106,619 (100.0)**  **n (column %)** | **Selected**  **80,360 (100.0)**  **n (column %)** | **Non-selected**  **26,259 (100.0)**  **n (column %)** | **P values** |
| --- | --- | --- | --- | --- |
| **Age,** years  18 to 59  60 to 69  70 to 79  ≥80 | 42,325 (39.7)  37,982 (35.6)  21,345 (20.0)  4,967 (4.7) | 32,420 (40.3)  29,234 (36.4)  15,639 (19.5)  3,067 (3.8) | 9,905 (37.7)  8,748 (33.3)  5,706 (21.8)  1,900 (7.2) | <0.001 |
| **Sex**  Male  Female | 40,586 (38.1)  66,033 (61.9) | 30,339 (37.8)  50,021 (62.2) | 10,247 (39.0)  16,012 (61.0) | <0.001 |
| **Ethnic groups**  Malay  Chinese  Indian  Bumiputera Sabah  Bumiputera Sarawak  Other ethnic groups | 70,378 (66.0)  15,015 (14.1)  7,651 (7.2)  8,252 (7.7)  3,382 (3.2)  1,941 (1.8) | 54,971 (68.4)  10,423 (13.0)  5,575 (6.9)  5,845 (7.3)  2,061 (2.6)  1,485 (1.8) | 15,407 (58.7)  4,592 (17.5)  2,076 (7.9)  2,407 (9.2)  1,321 (5.0)  456 (1.7) | <0.001 |

Chi-square tests were used to compare the proportions between selected and non-selected patients.
