# Supplementary material for: Prevalence of diabetic kidney disease and the associated factors among patients with type 2 diabetes in a multi-ethnic Asian country
Source: Sci Rep. 2024 Mar 25;14:7074. doi: 10.1038/s41598-024-57723-6 (PMC10963363; doi:10.1038/s41598-024-57723-6)
Supplement: Supplementary file 2 — Supplementary Table S2. [file 41598_2024_57723_MOESM2_ESM.docx]

**Supplementary Table S2: Characteristics of patients with diabetic kidney disease, n = 80,360**

| **Characteristics** | **No diabetic kidney disease**  **34,782 (43.3)**  **n (%)** | **Diabetic kidney disease**  **45,578 (56.7)**  **n (%)** | ***P* values** |
| --- | --- | --- | --- |
| **Age,** years  Mean ± standard deviation  18 to 59  60 to 69  70 to 79  ≥80 | 59.7 ± 10.7  16,127 (49.7)  12,719 (43.5)  5,192 (33.2)  744 (24.3) | 62.7 ± 11.1  16,293 (50.3)  16,515 (56.5)  10,447 (66.8)  2,323 (75.7) | <0.001  <0.001 |
| **Sex**  Male  Female | 12,079 (39.8)  22,703 (45.4) | 18,260 (60.2)  27,318 (54.6) | <0.001 |
| **Ethnic groups**  Malay  Chinese  Indian  Bumiputera Sabah  Bumiputera Sarawak  Other ethnic groups | 22,934 (41.7)  4,565 (43.8)  2,841 (51.0)  2,653 (45.4)  1,068 (51.8)  721 (48.6) | 32,037 (58.3)  5,858 (56.2)  2,734 (49.0)  3,192 (54.6)  993 (48.2)  764 (51.4) | <0.001 |
| **Current smoking**  Yes  No | 1,983 (41.4)  32,799 (43.4) | 2,805 (58.6)  42,773 (56.6) | 0.007 |
| **Duration of diabetes,** years  Median (interquartile range)  <5  5–10  >10 | 6.0 (7.0)  13,503 (50.4)  13,320 (44.3)  7,959 (33.8) | 8.0 (9.0)  13,280 (49.6)  16,715 (55.7)  15,583 (66.2) | <0.001  <0.001 |
| **Body mass index,** kg/m^2^ (n = 76,248)  Mean ± standard deviation  Underweight (<18.5)  Normal (18.5 to 22.9)  Overweight (23.0 to 27.4)  Obese (≥27.5) | 27.8 ± 5.3  496 (40.5)  4,847 (45.7)  12,009 (44.6)  15,847 (42.3) | 28.1 ± 5.4  728 (59.5)  5,770 (54.3)  14,908 (55.4)  21,643 (57.7) | <0.001  <0.001 |
| **Hypertension**  Yes  No | 29,447 (41.0)  5,335 (62.5) | 42,375 (59.0)  3,203 (37.5) | <0.001 |
| **Dyslipidaemia**  Yes  No | 31,758 (42.8)  3,024 (49.5) | 42,497 (57.2)  3,081 (50.5) | <0.001 |
| **Diabetic retinopathy**  Yes  No | 2,733 (32.8)  32,049 (44.5) | 5,601 (67.2)  39,977 (55.5) | <0.001 |
| **Diabetic foot ulcer**  Yes  No | 153 (20.7)  34,629 (43.5) | 586 (79.3)  44,992 (56.5) | <0.001 |
| **Nontraumatic lower-extremity amputation**  Yes  No | 79 (18.1)  34,703 (43.4) | 357 (81.9)  45,221 (56.6) | <0.001 |
| **Ischemic heart disease**  Yes  No | 1,355 (32.9)  33,427 (43.8) | 2,763 (67.1)  42,815 (56.2) | <0.001 |
| **Stroke**  Yes  No | 439 (31.0)  34,343 (43.5) | 975 (69.0)  44,603 (56.5) | <0.001 |
| **Diabetes treatment modality**  Lifestyle management only  Oral glucose-lowering drug (OGLD) only  Insulin only  OGLD and insulin | 1,237 (47.6)  25,376 (47.3)  826 (20.2)  7,343 (36.7) | 1,363 (52.4)  28,316 (52.7)  3,261 (79.8)  12,638 (63.3) | <0.001 |
| **Number of antihypertensive agents**  0  1  2  ≥3 | 6,412 (59.8)  11,103 (48.7)  10,994 (40.6)  6,273 (31.7) | 4,306 (40.2)  11,675 (51.3)  16,107 (59.4)  13,490 (68.3) | <0.001 |
| **Lipid-lowering agents**  Yes  No | 30,303 (42.9)  4,479 (46.1) | 40,337 (57.1)  5,241 (53.9) | <0.001 |
| **Antiplatelet agents**  Yes  No | 5,169 (34.5)  29,613 (45.3) | 9,807 (65.5)  35,771 (54.7) | <0.001 |
| **HbA1c,** % (n = 79,736)  Mean ± standard deviation  <7.0 %  7.0 to 8.0 %  >8.0 % | 7.65 ± 1.94  16,487 (47.3)  7,140 (43.5)  10,877 (38.2) | 7.99 ± 2.11  18,349 (52.7)  9,264 (56.5)  17,619 (61.8) | <0.001  <0.001 |
| **Systolic blood pressure,** mmHg (n = 79,950)  Mean ± standard deviation  <130 mmHg  130 to 139 mmHg  140 to 159 mmHg  160 to 179 mmHg  ≥180 mmHg | 134. 6 ± 14.7  12,324 (47.1)  10,114 (45.2)  10,547 (40.5)  1,515 (31.6)  150 (24.8) | 137.2 ± 16.0  13,852 (52.9)  12,248 (54.8)  15,465 (59.5)  3,280 (68.4)  455 (75.2) | <0.001  <0.001 |
| **Diastolic blood pressure,** mmHg (n = 79,944)  Mean ± standard deviation  <80 mmHg  80 to 89 mmHg  90 to 99 mmHg  100 to 109 mmHg  ≥110 mmHg | 77.8 ± 9.7  19,326 (42.0)  11,486 (45.9)  3,566 (43.7)  227 (33.9)  38 (33.6) | 76.9 ± 10.5  26,661 (58.0)  13,528 (54.1)  4,594 (56.3)  443 (66.1)  75 (66.4) | <0.001  <0.001 |
| **Blood pressure goal** (n = 79,938)  <140/80 mmHg  ≥140/80mmHg | 14,482 (44.4)  20,159 (42.6) | 18,130 (55.6)  27,167 (57.4) | <0.001 |
| **Blood pressure goal** (n = 79,938)  <130/80 mmHg  ≥130/80 mmHg | 9,121 (45.8)  25,520 (42.5) | 10,807 (54.2)  34,490 (57.5) | <0.001 |
| **LDL-cholesterol,** mmol/L (n = 71,037)  Mean ± standard deviation | 2.82 ± 1.07 | 2.82 ± 1.15 | 0.570 |
| **LDL-cholesterol goal** (n = 71,037)  <2.6 mmol/L  ≥2.6 mmol/L | 14,103 (43.3)  17,080 (44.4) | 18,447 (56.7)  21,407 (55.6) | 0.005 |
| **LDL-cholesterol goal** (n = 71,037)  <1.4 mmol/L  ≥1.4 mmol/L | 1,524 (38.9)  29,659 (44.2) | 2,391 (61.1)  37,463 (55.8) | <0.001 |
| **Triglyceride,** mmol/L (n = 78,989)  Mean ± standard deviation  <1.7 mmol/L  ≥1.7 mmol/L | 1.55 ± 0.90  23,167 (46.7)  11,088 (37.7) | 1.74 ± 1.06  26,434 (53.3)  18,300 (62.3) | <0.001  <0.001 |
| **HDL-cholesterol,** mmol/L (n = 71,422)  Mean ± standard deviation (overall) (n = 71,422)  Mean ± standard deviation (males) (n = 26,706)  Mean ± standard deviation (females) (n = 44,716)  >1.0 for males and >1.3 for females  ≤1.0 for males and ≤1.3 for females | 1.35 ± 0.39  1.24 ± 0.36  1.41 ± 0.40  19,085 (45.6)  12,221 (41.4) | 1.31 ± 0.41  1.20 ± 0.38  1.38 ± 0.41  22,807 (54.4)  17,309 (58.6) | <0.001  <0.001  <0.001  <0.001 |
