# Supplementary material for: Prevalence of diabetic kidney disease and the associated factors among patients with type 2 diabetes in a multi-ethnic Asian country
Source: Sci Rep. 2024 Mar 25;14:7074. doi: 10.1038/s41598-024-57723-6 (PMC10963363; doi:10.1038/s41598-024-57723-6)
Supplement: Supplementary file 3 — Supplementary Table S3. [file 41598_2024_57723_MOESM3_ESM.docx]

**Supplementary Table S3: Demographic comparisons between included and non-included patients in the multiple logistic regression analysis**

| **Characteristics** | **Total**  **80,360 (100.0)**  **n (column %)** | **Included**  **67,368 (100.0)**  **n (column %)** | **Non-included**  **12,992 (100.0)**  **n (column %)** | **P values** |
| --- | --- | --- | --- | --- |
| **Age,** years  18 to 59  60 to 69  70 to 79  ≥80 | 32,420 (40.3)  29,234 (36.4)  15,639 (19.5)  3,067 (3.8) | 27,684 (41.1)  24,640 (36.6)  12,721 (18.9)  2,323 (3.4) | 4,736 (36.5)  4,594 (35.4)  2,918 (22.5)  744 (5.6) | <0.001 |
| **Sex**  Male  Female | 30,339 (37.8)  50,021 (62.2) | 25,218 (37.4)  42,150 (62.6) | 5,121 (39.4)  7,871 (60.6) | <0.001 |
| **Ethnic groups**  Malay  Chinese  Indian  Bumiputera Sabah  Bumiputera Sarawak  Other ethnic groups | 54,971 (68.4)  10,423 (13.0)  5,575 (6.9)  5,845 (7.3)  2,061 (2.6)  1,485 (1.8) | 47,289 (70.2)  8,288 (12.4)  4,676 (6.9)  4,680 (6.9)  1,196 (1.8)  1,239 (1.8) | 7,682 (59.1)  2,135 (16.4)  899 (6.9)  1,165 (9.0)  865 (6.7)  246 (1.9) | <0.001 |

Chi-square tests were used to compare the proportions between included and non-included patients.
